# Windshield wipers on connected vehicles produce high-accuracy rainfall maps

**DOI:** 10.1038/s41598-018-36282-7

**Published:** 2019-01-17

**Authors:** Matthew Bartos, Hyongju Park, Tian Zhou, Branko Kerkez, Ramanarayan Vasudevan

**Affiliations:** 1 Department of Civil and Environmental Engineering, University of Michigan, Ann Arbor, MI 48109 United States; 2 Department of Mechanical Engineering, University of Michigan, Ann Arbor, MI 48109 United States

## Abstract

Connected vehicles are poised to transform the field of environmental sensing by enabling acquisition of scientific data at unprecedented scales. Drawing on a real-world dataset collected from almost 70 connected vehicles, this study generates improved rainfall estimates by combining weather radar with windshield wiper observations. Existing methods for measuring precipitation are subject to spatial and temporal uncertainties that compromise high-precision applications like flash flood forecasting. Windshield wiper measurements from connected vehicles correct these uncertainties by providing precise information about the timing and location of rainfall. Using co-located vehicle dashboard camera footage, we find that wiper measurements are a stronger predictor of binary rainfall state than traditional stationary gages or radar-based measurements. We introduce a Bayesian filtering framework that generates improved rainfall estimates by updating radar rainfall fields with windshield wiper observations. We find that the resulting rainfall field estimate captures rainfall events that would otherwise be missed by conventional measurements. We discuss how these enhanced rainfall maps can be used to improve flood warnings and facilitate real-time operation of stormwater infrastructure.

## Introduction

Accurate rainfall measurements are essential for the effective management of water resources^1^. Historical rainfall records are used extensively in the design of water infrastructure^2^, while at finer scales, real-time rainfall measurements are an integral component of flood forecasting systems^3^. Despite the central role that precipitation measurements play in the design and operation of water infrastructure, current methods for measuring precipitation often do not provide the spatial resolution or measurement certainty required for real-time applications^3^. As the demand for real-time precipitation data increases, new sensing modalities are needed to address deficiencies found in conventional data sources.

The need for high-resolution precipitation estimates is perhaps best illustrated by the problem of urban flash flooding. Flooding is the number one cause of natural disaster fatalities worldwide, with flash floods accounting for a majority of flooding deaths in developed countries^4^. Despite the risks posed by flash flooding, there is “no existing model [that is] capable of making reliable flash flood forecasts in urban watersheds”^3^. Flash flood forecasting is to a large extent hindered by a lack of high-resolution precipitation data, with spatial resolutions of <500 m and temporal resolutions of 1–15 minutes required for urban areas^5,6^.

Contemporary rain measurement technologies—such as stationary rain gages and weather radar—struggle to achieve the level of precision necessary for flash flood forecasting. While rain gages have long served as a trusted source of surface-level precipitation measurements^7^, they often fail to capture the spatial variability of rain events, especially during convective storms^8–10^. This inability to resolve spatial patterns in rainfall is made worse by the fact that the number of rain gages worldwide is rapidly declining^1^. Weather radar is a useful tool for capturing the spatial distribution of rainfall. However, radar-rainfall estimates are subject to large spatial and temporal uncertainties^11–14^. Additionally, weather radar tends to show systematically large biases for major flood events, and may perform poorly for small watersheds^6^, making urban flood forecasting problematic.

The rise of connected and autonomous vehicles offers an unprecedented opportunity to enhance the density of environmental measurements^15,16^. While dedicated sensor networks are expensive to deploy and maintain, fleets of connected vehicles can capture real-time data at fine spatial and temporal scales through the use of incidental onboard sensors. With regard to rainfall measurement, windshield wiper activity offers a novel means to detect the location and timing of rainfall with enhanced precision. When used in conjunction with modern signal processing techniques, wiper-based sensing offers several attractive properties: (i) vehicles achieve vastly improved coverage of urban areas, where flood monitoring is important; (ii) windshield wiper intensity is easy to measure and requires little overhead for processing (as opposed to video or audio data); and (iii) vehicle-based sensing can be readily scaled as vehicle-to-infrastructure communication becomes more widespread. Moreover, many new vehicles come equipped with optical rain sensors that enable direct measurement of rainfall intensities. When paired with data assimilation techniques, these sensors may enable even higher-accuracy estimation of rainfall fields compared to wipers alone.

While a small number of studies have investigated vehicle-based precipitation measurements, the results of these studies are strictly based on simulated wiper data instead of real measurements. As such, the premise that windshield wiper data can be used to improve rainfall estimates has never been verified using a large real-world dataset. Hill (2015) combines simulated binary (wet/dry) rainfall sensors with weather radar observations to generate improved areal rainfall estimates, which are then validated against rainfall fields produced by interpolation of tipping-bucket rain gages^15^. Similarly, Haberlandt (2010) combines simulated vehicle wiper measurements with rain gage observations to improve rainfall field estimates, and then validates the resulting product against weather radar^16^. Although these studies highlight the potential for vehicle-based measurements to improve the spatial and temporal resolution of rainfall estimates, their findings have not yet been validated using data from real-world connected vehicles.

**Figure 1:**
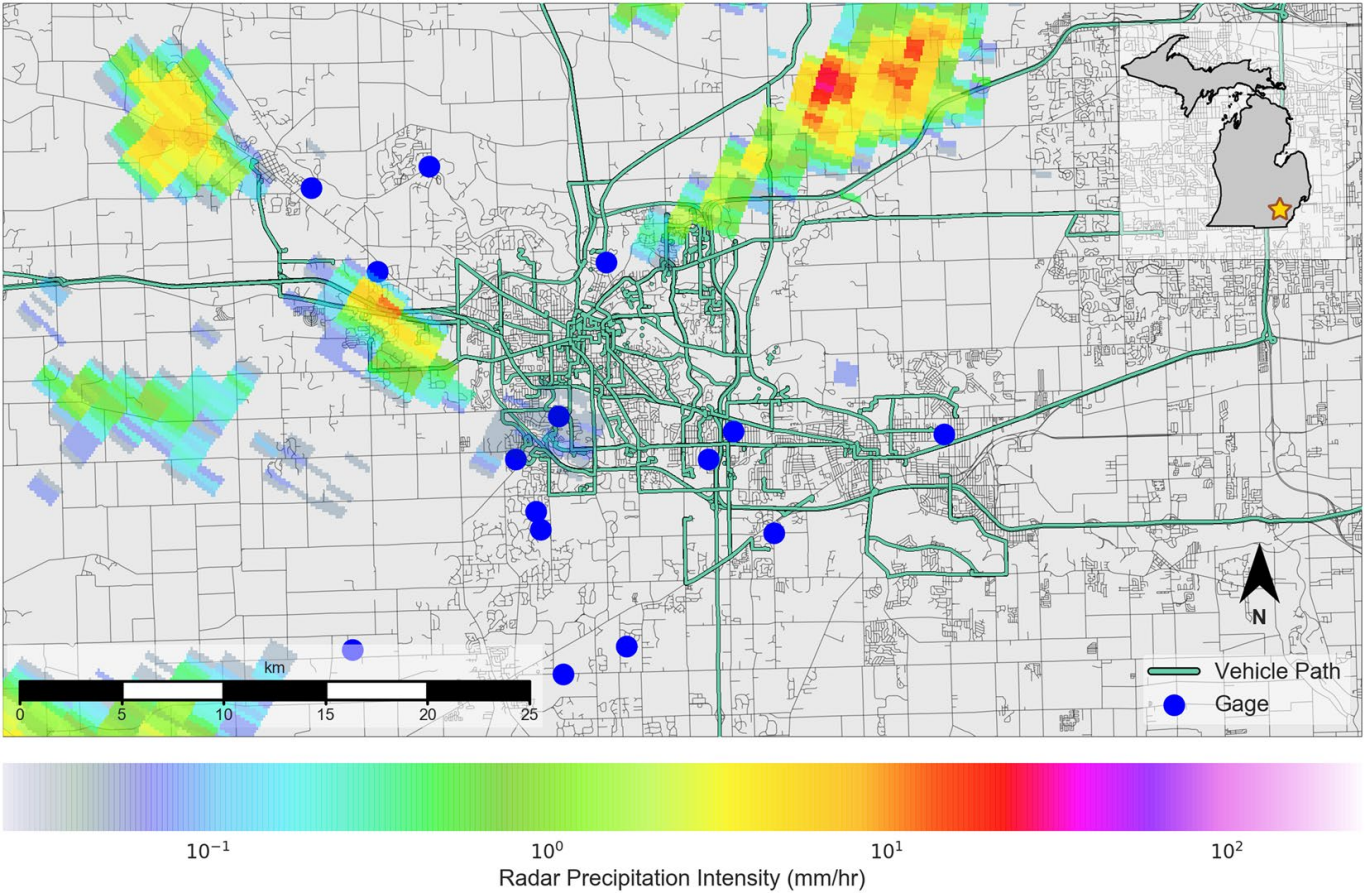
Overview of the study area on June 12, 2014. Blue circles represent rain gages. Vehicle paths are shown as green lines, while roads are shown in gray. A radar overlay shows the average precipitation intensity as estimated by radar. The map is produced with a custom script using the Python programming language (Python 3.6: https://www.python.org). See data access links for code used to generate this map.

To address these challenges, this study leverages windshield wiper measurements collected from nearly 70 vehicles to produce corrected rainfall maps (see Fig. 1 for a description of the study area and data sources). In the first part of this paper, we demonstrate that windshield wiper measurements offer a reliable indicator of rainfall by comparing wiper measurements against dashboard camera footage that indicates the ground truth binary rainfall state (raining/not raining). In the second part of this paper, we develop a Bayesian data fusion procedure that combines weather radar with vehicle-based wiper measurements to produce an updated probabilistic rainfall field map. We validate this novel data product by showing that it is more effective than the original radar data at predicting the binary rainfall state. Finally, we discuss how these enhanced rainfall maps can be used to improve flood warnings and facilitate real-time operation of stormwater infrastructure.

## Results

### Windshield wipers improve binary rainfall detection

Windshield wiper measurements enhance rainfall estimation by enabling greater certainty about the timing and location of rainfall. While wiper intensity on its own is generally a poor predictor of rainfall intensity (see Figure S1 in the Supplementary Information), we find that wiper status (on/off) is a stronger predictor of binary rainfall state than either radar or gage-based measurements. This result suggests that vehicle-based measurements can be used to validate and correct rainfall fields derived from conventional data sources.

**Figure 2:**
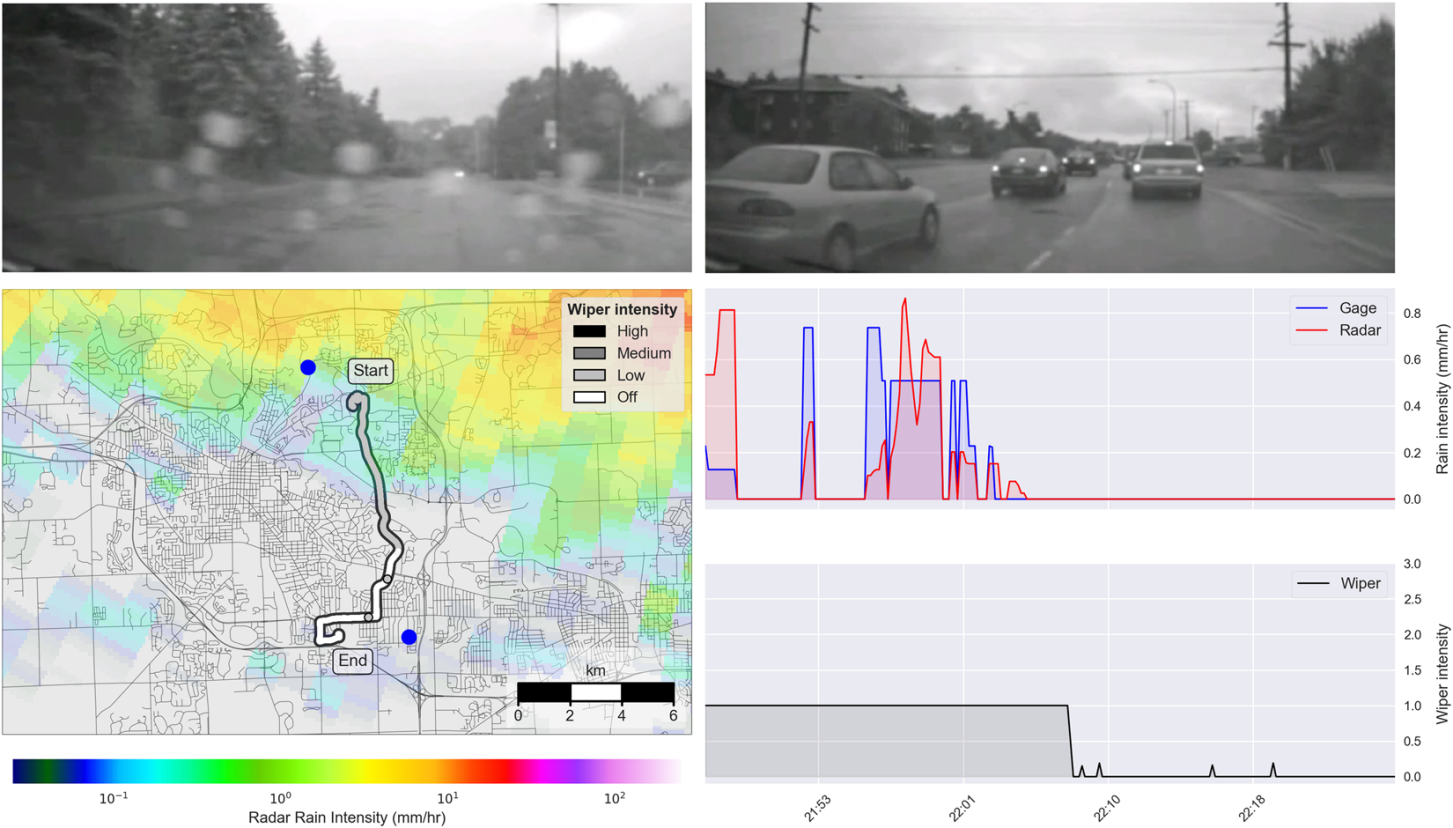
Analysis of a single vehicle trip occurring from 21:46–22:26 on August 11, 2014. The top two panels show video footage during the rainy (left) and dry (right) segments of the trip. The bottom left panel shows a map of the vehicle’s trip, with the wiper intensity indicated by color. A radar overlay shows the average rainfall intensity over the 40-minute time period. Blue circles represent the gages nearest to the vehicle path. The two bottom right panels show the precipitation intensity as estimated by radar and gage measurements (center), and the 1-minute average wiper intensity (bottom). Photographs are reproduced with permission from the University of Michigan Transportation Research Institute. The map is produced with a custom script using the Python programming language (Python 3.6: https://www.python.org). See data access links for code used to generate this map.

Wiper measurements provide a more accurate indicator of binary rainfall state than either radar or gage measurements. We determine the binary classification performance for each technology (gages, radar and wipers) by comparing the measured rainfall state with co-located dashboard video footage. Dashboard video is taken to represent the ground truth, given that the presence or absence of rainfall can readily be determined by visually inspecting the windshields for raindrops. Figure 2 shows an example of co-located radar, gage, wiper and camera measurements for a single vehicle trip. The top two frames show dashboard camera footage collected over the course of the vehicle trip. Rainfall is visible during the first half of the trip (top left) while no rain can be seen during the second half of the trip (top right). The map (bottom left) shows the path of the vehicle along with (i) the reported wiper intensity, (ii) the average radar rainfall intensity during the trip, and (iii) the two nearest rain gages. Two time series (right) compare radar and gage measurements of rainfall intensity near the vehicle’s location (center right) with reported wiper intensity (bottom right). The binary classification performance for each data source is assessed by manually labeling the ground truth rainfall state based on the dashboard camera footage, and then comparing these labels with the binary rainfall state predicted by co-located wiper, radar and rain gage data sources.

Comparing radar, gage, and wiper measurements with co-located vehicle footage across three storm events, we find that wiper status is the best estimator of binary rainfall state, with a true positive rate (TPR) of 93.1%, and a true negative rate (TNR) of 98.2%. By comparison, weather radar achieves a smaller TPR of 89.5%, while stationary gages show a much smaller TPR of 44.5% (see Table 1). These results can partly be explained by the superior spatial and temporal resolution of the wiper measurements. Wipers detect intermittent changes in rainfall at a temporal resolution on the order of seconds, while radar and gage measurements can only detect the average rate over a 5-minute period. When ground truth camera observations are collected at a 3-second temporal resolution, the benefit of wiper measurements over radar measurements becomes even more pronounced, with a TPR advantage of 5.2%, a TNR advantage of 7.7%, and an overall wiper TPR of 97.0% (see the supplementary note on factors affecting binary detection performance). The results of this analysis suggest that conventional rainfall measurement technologies can be enhanced through the inclusion of vehicle-based measurements.

**Table 1:** Classification performance of each rainfall measurement technology.

Metric	Gage	Radar	Wiper
True Positive Rate (%)	44.5	89.5	93.1
True Negative Rate (%)	96.7	97.5	98.2

The true positive rate indicates the percentage of instances where the given technology successfully detects rainfall when rainfall is actually occurring. The true negative rate indicates the percentage of instances where the technology does not detect rainfall when rainfall is not occurring.

### Assimilation of wiper data yields corrected rainfall maps

**Figure 3:**
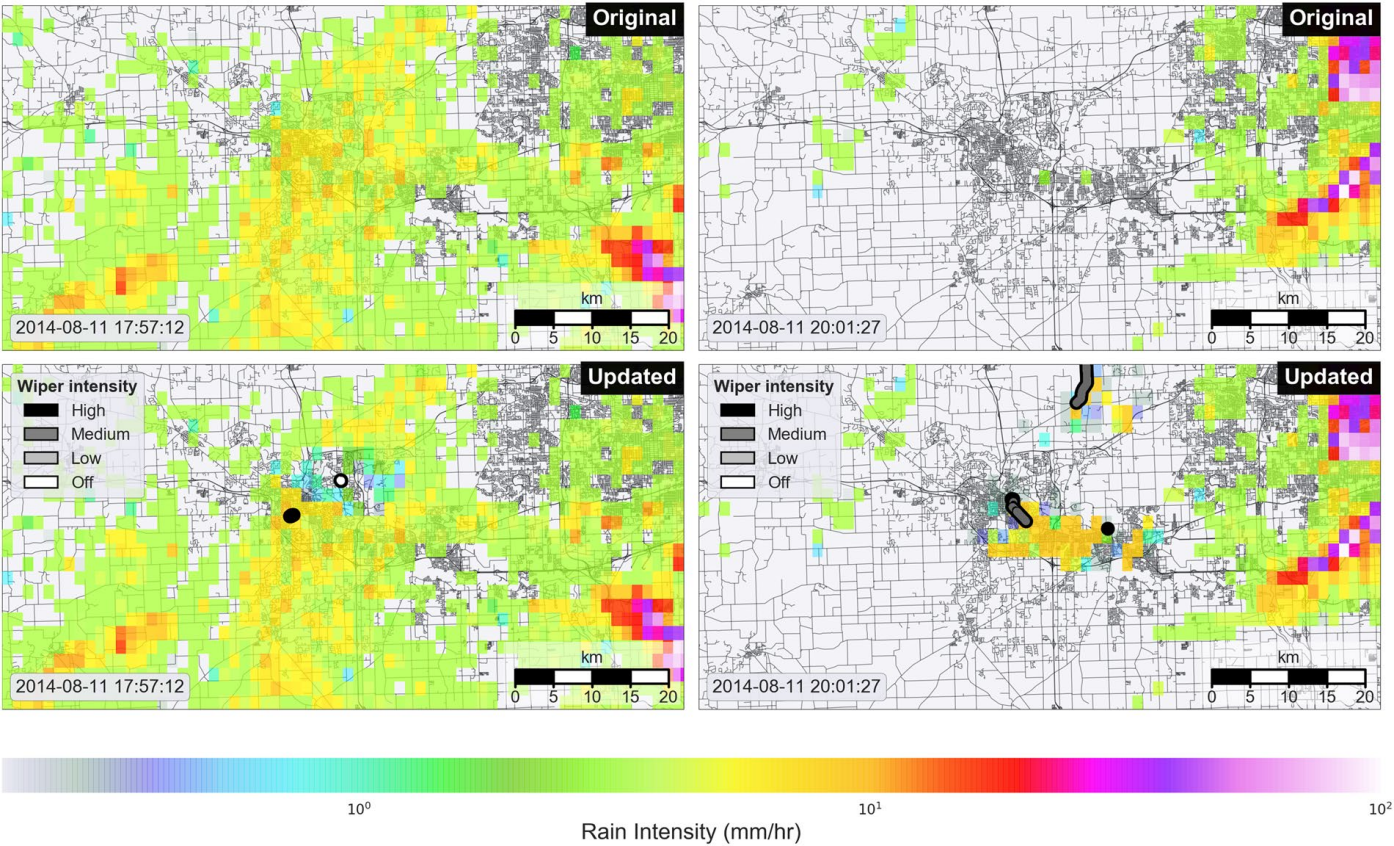
Original and updated rainfall maps. Top (left and right): Original prior weather radar rainfall intensity map. Radial radar scans have been resampled to a 1 km grid to ensure computational tractability. Bottom (left and right): updated posterior rainfall intensity map, combining radar data with wiper measurements using the Bayesian filter. In the bottom left panel, a “hole” in the rainfall field occurs when a vehicle detects no rain in a location where radar alone estimated rain. In the bottom right panel, vehicles detect rainfall where radar previously did not detect rainfall. The map is produced with a custom script using the Python programming language (Python 3.6: https://www.python.org). See data access links for code used to generate this map.

Based on the observation that wiper measurements are a strong binary predictor of rainfall, we develop a Bayesian filtering framework that combines radar rainfall estimates with wiper observations to generate corrected rainfall maps. Radar is first used to estimate a prior distribution of rainfall intensities. This prior is then updated with wiper observations to produce a corrected rainfall intensity field that better captures the binary rainfall state. The results of this filtering procedure are demonstrated in Fig. 3, which shows the original rainfall intensity field (top) along with the corrected rainfall intensity field (bottom). Vehicle paths are shown (bottom) to highlight the effect of wiper measurements on the posterior rainfall intensity distribution. In cases where both radar and wipers agree on the binary rainfall state, the rainfall intensity field remains unchanged. For example, when the wiper and radar intensities are both nonzero (as seen in the bottom-left panel, leftmost vehicle), the posterior rainfall intensity is simply equal to the prior rainfall intensity. In other cases, vehicles detect no rainfall in regions where radar had previously estimated rainfall (bottom-left panel, rightmost vehicle). In these cases, the Bayesian filter reduces the intensity of the rainfall field within the proximity of the vehicle. Conversely, in the case where vehicles detect rainfall in regions where little to no rainfall was observed in the original dataset (right panel), the Bayesian filter amplifies the rainfall intensity field within the vicinity of the vehicle, resulting in a new rainfall intensity distribution that better represents the binary rainfall state. The predicted rainfall intensity depends on both the wiper measurement and the intensity of the radar rainfall prior within the neighborhood of the vehicle. Thus, vehicles located near a prior rainfall front (bottom-right panel, center of frame) will have a larger effect on the posterior rainfall intensity than vehicles located far away from a prior rainfall front (bottom-right panel, top of frame). For a more detailed view of the evolution of the rainfall field under both the original and corrected data sets, refer to Video S1.

**Figure 4:**
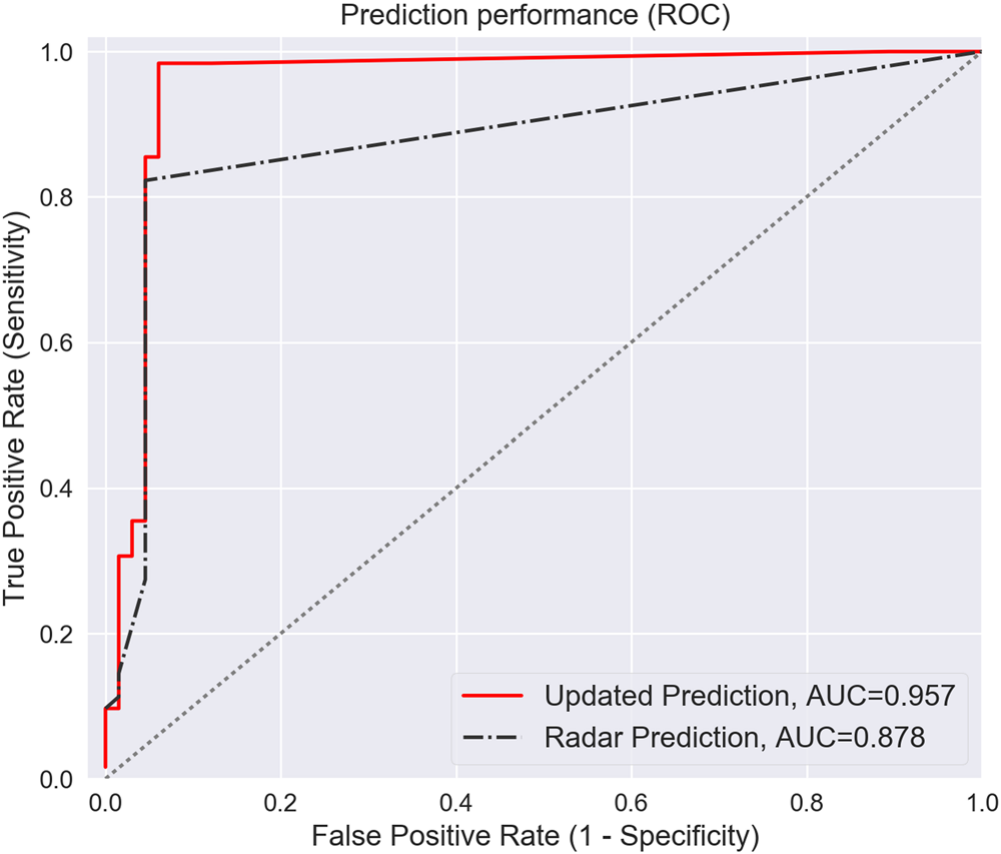
Binary classification performance of the updated rainfall product. Receiver operator characteristic (ROC) curves indicate the rainfall state prediction accuracy for the original radar estimate and the updated (wiper-corrected) data product. The area under the curve (AUC) measures overall classification performance.

The wiper-corrected rainfall field predicts the binary rainfall state with greater accuracy than the radar-only data product. To validate the wiper-corrected rainfall field, we use an iterated “leave-one-out” approach, in which an updated rainfall field is generated while excluding a vehicle, and the resulting data product is compared against the measured rainfall state of the omitted vehicle. Repeating this process for each vehicle yields the receiver operator characteristics shown in Fig. 4. These curves map the relationship between the TPR and TNR for both the original rainfall field (radar only) and the corrected rainfall field (radar and wiper). Curves located closer to the upper-left corner (i.e. those with a larger area under the curve) exhibit the best performance, given that they have a large true positive rate and a small false negative rate. Based on these curves, it can be seen that the corrected data product performs consistently better than the original radar product at predicting the presence or absence of rain, with a TPR and TNR close to unity. The overall performance of the updated rainfall product—as measured by the area under the curve (AUC)—is roughly 0.957, compared to only 0.878 for the original radar data. These results confirm that inclusion of vehicle-based measurements enables improved prediction of the underlying rainfall field.

## Discussion

The enhanced rainfall maps developed in this study have the potential to assist in the real-time operation of transportation and water infrastructure. In particular, high accuracy rainfall field estimates will enable improved prediction of flash floods in urban centers, and will help to inform real-time control strategies for stormwater systems. As mentioned previously, flash flood forecasting is contingent on high-resolution areal rainfall estimates, with accurate measurements on the order of 500 m or finer required for forecasting in urban areas. By enabling real-time validation and filtering of radar rainfall estimates, vehicle-based sensors may help fill measurement gaps and improve the prediction of flood events near roadways. Monitoring of roadways is especially important given that in the US, roughly 74% of flood fatalities are vehicle related^4^. As connected and autonomous vehicles become more widely adopted, the spatial coverage and measurement certainty of this new sensing modality will be even further enhanced.

In addition to assisting with flash flood response, high-precision rainfall data products may one day inform the operation of new “smart” water infrastructure. Recent work has highlighted the potential of “smart” water systems to mitigate water hazards through real-time control of distributed gates, valves and pumps^17–21^. When informed by accurate and timely data, these systems can significantly reduce operating costs, prevent combined sewer overflows, and halt the degradation of aquatic ecosystems by adaptively reconfiguring water infrastructure in real time^17,18^. However, recent findings suggest that optimal control strategies for “smart” water systems are highly sensitive to the location, timing and intensity of rainfall inputs^22^. In this regard, the wiper-corrected rainfall product presented in this study may help to enable more fine-grained control of water infrastructure by reducing uncertainty in conventional rainfall field estimates.

While this work evaluates the updated rainfall product in terms of its ability to predict the binary rainfall state, future work should use vehicle-based sensors to further validate and improve the predicted rainfall intensity. Currently, visual inspection of the ground truth data source (camera footage) only allows for verification of the binary rainfall state and not the predicted rainfall intensity. Other potential sources of ground truth rainfall intensity, such as stationary rain gages, are also problematic. While rain gages provide an independent source of rainfall intensity data, they are only able to produce estimates of rainfall accumulation at point locations every 5 minutes, and are often far removed from the nearest vehicle path. Moreover, as shown in Table 1, gages are by far the poorest predictor of the binary rainfall state among all data sources considered. These issues raise questions as to the appropriateness of rain gages as a source of ground truth rainfall intensity data. With these issues in mind, a natural extension of the work presented in this paper could use other vehicle-based sensors to better estimate the rainfall intensity at each vehicle’s location. Drawing on dashboard camera footage, object detection techniques could be used to isolate and count raindrops on the windshield of each vehicle. The volume of rainfall deposited over each wiper interval may then be estimated, thereby yielding an estimate of rainfall intensity at the vehicle’s location. Similarly, many newer vehicles feature optical rain sensors that are capable of measuring precipitation rate directly. When combined with the Bayesian sensor fusion framework presented in this study, these sensors could enhance the accuracy of the estimated rainfall intensity field. While outside the scope of this work, these techniques represent promising directions for future research and should be considered in subsequent studies.

## Conclusions

This study generates enhanced probabilistic rainfall maps by combining conventional radar-based precipitation fields with ubiquitous windshield wiper measurements from almost 70 unique vehicles. We find that while windshield wiper intensity is a poor predictor of rainfall intensity, wiper activity is a stronger predictor of binary rainfall state than conventional radar and gage-based data sources. With this result in mind, we develop a novel Bayesian filtering framework that combines a radar-based rainfall prior with binary windshield wiper observations to produce an updated rainfall map. We find that the Bayesian filtering process is effective at detecting changes in the rainfall field that conventional measurement technologies may otherwise miss. We validate the updated rainfall data product by assessing its ability to reproduce the binary rainfall state anticipated by an omitted vehicle. Based on this analysis, we find that the corrected rainfall field is better at predicting the binary rainfall state than the original radar product. As connected vehicles become more widespread, the ubiquitous sensing approach proposed by this study may one day help to inform real-time warning and control systems for water infrastructure by providing fine-grained estimates of the rainfall field.

## Materials and Methods

### Evaluating vehicle-based measurements

In the first part of this study, we assess the degree to which windshield wiper activity serves as a proxy for both rainfall intensity and binary rainfall state. First, wiper measurements are compared against conventional rainfall measurement technologies to determine if there is a direct relationship between wiper intensity and rain intensity. Next, we assess the degree to which each data source reflects the ground truth rainfall state by comparing measurements from all three sources (gages, radar and wipers) with vehicle-based video footage. Video footage provides instantaneous visual confirmation of the rainfall state (raining or not raining), and is thus taken to represent the ground truth. We characterize the binary classification performance of each technology in terms of its true positive and true negative rates.

To ensure that our analysis is computationally tractable, we isolate the study to a subset of three storms in 2014. We assess the validity of our procedure for storms of different magnitudes by selecting a large storm (2014-08-11), a medium-sized storm (2014-06-28) and a small storm (2014-06-12). Storms are selected during the summertime months to avoid conflating rainfall measurements with snow measurements. The year 2014 is chosen because it is the year for which the greatest number of vehicles are available. Unless otherwise specified, data are co-located using a nearest neighbor search. For comparison of wiper and gage readings, we select only those gages within a 2 km range of any given vehicle.

### Data sources

We consider four data sources: (i) stationary rain gages, (ii) weather surveillance radar, (iii) vehicle windshield wiper data, and (iv) vehicle dashboard camera footage. We provide a brief description of each data source here:

Gage data are obtained from personal weather stations maintained by the Weather Underground^23^. Within the city of Ann Arbor (Michigan), Weather Underground hosts 21 personal weather stations, each of which yield rainfall estimates at a time interval of approximately 5 minutes. Locations of gages are indicated by blue circles in Fig. 1. Although verified gage data from the National Weather Service (NWS) and the National Oceanic and Atmospheric Administration (NOAA) are available, Weather Underground gages are selected because (i) NOAA and NWS each maintain only a single gage in the city of Ann Arbor, meaning that intra-urban spatial variations in precipitation intensity cannot be captured, and (ii) the temporal resolution of NOAA and NWS gages are relatively coarse for real-time applications (with NOAA offering a maximum temporal resolution of 15 minutes and NWS offering a maximum temporal resolution of 1 hour).

Weather radar observations are obtained from NOAA’s NEXRAD Level 3 Radar product archive^24^. We use the “Instantaneous Precipitation Rate” data product (listed as variable code 176 in the NEXRAD Level 3 archive^25^). Radar precipitation estimates are obtained at a temporal resolution of 5 minutes, and a spatial resolution of 0.25 km by 0.5 degree (azimuth). Radar station KDTX in Detroit is used because it is the closest radar station to the City of Ann Arbor. Radial radar scans are interpolated to cartesian coordinates using a nearest neighbor approach.

Vehicle-based wiper intensities are obtained from the University of Michigan Transportation Research Institute (UMTRI) Safety Pilot Model Deployment database^26^. For each vehicle, this dataset includes time series of latitude, longitude, and windshield wiper intensity at a temporal resolution of 2 milliseconds. Windshield wiper intensity is given on an ordinal scale from 0 to 3, with 0 indicating that the wiper is turned off, 1 representing the lowest wiper intensity, and 3 representing the highest wiper intensity. A wiper reading of 4 indicates that the vehicle’s “mister” is activated, distinguishing between wiper use for rain removal and wiper use for windshield cleaning. For this study, wiper usage for cleaning (i.e. wiper mode 4) was filtered out before the analysis. Note that wiper intensity codes are based on electrical signals generated by the wiper itself, meaning that no manual wiper mode classification is needed. For the year 2014, 69 unique vehicles are available in the UMTRI dataset. However, typically less than ten vehicles are active at any given time during the observation period. Vehicles with no sensor output or invalid readings were removed from the dataset prior to the analysis (see the supplementary note for more details). Other sources of human error (such as accidentally turning wipers on), are captured by the true positive and true negative rates included in Tables 1 and S1.

Camera observations are also obtained from the UMTRI vehicle database^26^. Located on the inside of each vehicle, cameras provide streaming video footage of the windshield, side-facing windows, rear-facing windows, and the driver. For the purposes of validation, we use the front-facing windshield camera. Camera frames are manually inspected for rain drops striking the windshield. Time intervals where rain is observed are classified as “raining”; similarly time intervals where no new droplets are observed are classified as “not raining”. Manual inspection and labeling of the video data was performed independently by two reviewers to ensure robustness.

### A Bayesian filtering framework

In the second part of this study, we develop a Bayesian filtering framework that combines binary wiper observations with radar-based rainfall intensity measurements to generate corrected rainfall maps. In simple terms, the Bayesian filter generates an updated rainfall field, in which binary (on/off) wiper measurements adaptively correct the underlying radar rainfall field. Windshield wiper status is taken to represent a measurement of the ground truth binary rainfall state, given that it is a better predictor of the binary rainfall state than radar- or gage-based measurements. Under this framework, four distinct cases are possible. If both the wiper and radar measure precipitation, the radar reading is taken to be correct, and the original rainfall field remains the same. Similarly, if neither the wiper nor the radar measure precipitation, the radar rainfall field remains zero. However, if the radar measures precipitation at a target location and the wiper does not, then the filter will update the rainfall field such that rainfall intensity is reduced within the proximity of the vehicle (with a decay pattern corresponding to the Gaussian kernel and an intensity of zero at the location of the wiper reading). Similarly, if the wiper measures precipitation, but the radar measures no precipitation, the rainfall intensity will be increased within the proximity of the vehicle (by combining the local distribution of the radar rainfall prior with a point estimate of rainfall intensity based on the wiper intensity). In our implementation, provided that no other information is available, this point estimate is generated using the empirical rainfall intensity distribution associated with the given wiper intensity. The empirical rainfall intensity distributions associated with each wiper intensity are shown in Figure S1 in the Supplementary Information.

Note that while wiper intensity by itself does not exhibit a strong correlation with rainfall intensity, the Bayesian filter uses both wiper and radar measurements to generate the posterior rainfall intensity estimate. In other words, the posterior rainfall intensity at the vehicle’s location is a probabilistic estimate that depends on both the wiper-based estimate and the local prior intensity within the neighborhood of the vehicle. Thus, a nonzero wiper measurement located far away from a radar rainfall front will result in a smaller posterior intensity than one located near a radar rainfall front (as discussed in the results section and shown in Fig. 3). The relative contribution of the wiper measurement and radar prior are controlled using a weighting parameter representing the user’s trust in each data source. This probabilistic assimilation of data sources helps to reduce the uncertainty associated with using the wiper intensity to estimate rainfall intensity. It should be noted that other methods for obtaining a point estimate of rainfall intensity are possible—such as choosing the closest nonzero intensity in the radar rainfall prior. For newer vehicles equipped with rain sensors, the rainfall intensity can also be measured directly using the sensor output. As mentioned in the discussion section, however, it is currently difficult to evaluate the relative accuracy of these approaches, given the lack of reliable ground truth rainfall intensity data at the appropriate spatial and temporal scales.

A more formal description of the filtering framework is given here in terms of a noisy sensor model (for additional details, see Park *et al.* (2018)^27^). Consider a noisy sensor model in which each sensor produces a binary measurement given a target state. The target state is represented as a random tuple ***z*** = (*q*, ***I***) where *q* is a location state (e.g. the latitude and longitude at the target), and ***I*** is an information state (e.g. the precipitation intensity at the target) with all the random quantities indicated by bold italics. We denote by *M*_*t*_ the event that sensors correctly measure the intensity, and by ${\bar{M}}_{t}$ the event that sensors fail to measure the intensity correctly. The joint measurement likelihood at any time *t* is given by:

1 $$p({M}_{t}|{\boldsymbol{z}},{x}_{t})$$

where *x*_*t*_ represents the locations of the sensors at time *t*. Equation 1 yields the probability distribution of precipitation intensity measurement at *q* by sensors at *x*_*t*_. The expected value of Equation 1 with respect to ***I*** is equivalent to the rainfall intensity experienced at the location *q*. Because the effective range of the wipers is limited, we account for the probability of detection as a function of the distance between the sensor and the target. We denote by *D*_*t*_ the event that sensors detect the target, and by ${\bar{D}}_{t}$ the event that sensors fail to detect the target at time *t*. The probability of detecting a target located at *q* by sensors located at *x*_*t*_, *p*(*D*_*t*_|*q*, *x*_*t*_), is taken to decay with increasing distance to the sensor. Using the law of total probability, the conditional probability of a correct measurement is then given by:

2 $$p({M}_{t}|{\boldsymbol{z}},{x}_{t})=p({M}_{t}|{\boldsymbol{z}},{D}_{t},{x}_{t})p({D}_{t}|q,{x}_{t})+p({M}_{t}|{\boldsymbol{z}},{\bar{D}}_{t},{x}_{t})p({\bar{D}}_{t}|q,{x}_{t})$$

where *D*_*t*_ is conditionally independent of ***I*** when conditioned on *q*. For example, consider *x*_*t*_ = (0, 0), and *q* = (*q*_1_, *q*_2_). If the decay function is taken to be a 2D Gaussian centered at *x*_*t*_ with covariance matrix σ**I** where **I** is a 2 by 2 identity matrix, then:

3 $$p({D}_{t}|q,{x}_{t})={\tilde{\eta }}_{t}\frac{1}{2\pi {\sigma }^{2}}\exp \,(-\frac{{q}_{1}^{2}+{q}_{2}^{2}}{2{\sigma }^{2}})$$

where ${\tilde{\eta }}_{t}$ is a normalization constraint. If the target is *not* detected (i.e., ${\bar{D}}_{t}$), then the measurement is assumed to be unreliable, and the likelihood, $p({M}_{t}|{\boldsymbol{z}},{\bar{D}}_{t},{x}_{t})$, is modeled using a prior distribution. If there is no prior information available, the function is modeled using a uniform distribution. Now let *b*_*t*_ (***z***) represent the posterior probability of the precipitation intensity given a target location *q* at time *t*. Using Bayes’ Theorem, *b*_*t*_ (***z***) can be formulated:

4 $${b}_{t}\,({\boldsymbol{z}})={\eta }_{t}\,p({M}_{t}|{\boldsymbol{z}},{x}_{t}){b}_{t-1}({\boldsymbol{z}}),\,t=1,2,\ldots $$

where *η*_*t*_ is a normalization constant and *b*_0_ is uniform if no information is available at *t* = 0. This filtering equation forms the basis of the rainfall field updating algorithm. To reduce computational complexity, the filtering operation is implemented using a Sequential Importance Resampling (SIR) Particle Filter^28^.

The results of the Bayesian sensor fusion procedure are evaluated by determining the proportion of instances where the combined data product is able to predict the binary rainfall state. We characterize the true and false positive rates for the largest storm event (2014-08-11) using an iterated “leave-one-out” cross-validation approach. First, a single vehicle is removed from the set of vehicles. The Bayesian update procedure is then executed using all vehicles except the excluded vehicle, and an updated rainfall map is generated. Next, the rainfall states predicted by the corrected rainfall field (radar and wiper) and the original rainfall field (radar only) are compared against the rainfall states predicted by the omitted vehicle. The performance of each data product is evaluated based on its ability to reproduce the binary rainfall state observed by the omitted vehicle. Performing this process iteratively yields the true and false positive rates for both the original (radar only) and updated (radar and wiper) rainfall fields. This procedure is repeated for each vehicle in the set of vehicles to generate Receiver-Operator Characteristic (ROC) curves, which characterize the true and false positive rates across an ensemble of simulations.

## Electronic supplementary material

Supplementary Information

Supplementary Video S1

## Data Availability

Code and data for this study are available at: github.com/kLabUM/vehicles-as-sensors.
