# Supplementary material for: Windshield wipers on connected vehicles produce high-accuracy rainfall maps
Source: Sci Rep. 2019 Jan 17;9:170. doi: 10.1038/s41598-018-36282-7 (PMC6336807; doi:10.1038/s41598-018-36282-7)
Supplement: Supplementary file 1 — Supplementary Information [file 41598_2018_36282_MOESM1_ESM.pdf]

Supplementary Information for
Windshield wipers on connected vehicles produce high-accuracy rainfall maps

Matthew Bartos[1,+], Hyongju Park[2,+], Tian Zhou[2], Branko Kerkez[1,*], and Ramanarayan Vasudevan[2]

[1]Department of Civil and Environmental Engineering, University of Michigan, Ann Arbor, MI, 48109, United States
[2]Department of Mechanical Engineering, University of Michigan, Ann Arbor, MI, 48109, United States
[*]bkerkez@umich.edu
[+]these authors contributed equally to this work

Supplementary note on binary detection performance

Binary detection performance is sensitive to a number of factors, including the temporal resolution of the ground truth data and the configuration of wiper sensors. While these factors can affect the magnitude of binary classification performance, under all scenarios considered, wiper measurements are a better detector of the binary rainfall state than either radar or gage measurements.

Binary detection performance can be affected by the temporal resolution at which the ground truth data is collected. To ensure robustness, labeling of vehicle footage was performed independently by two reviewers. The first reviewer labeled the observed rainfall state for each vehicle over all three days of the study period (2014-06-12, 2014-06-28, 2014-08-11) at a temporal interval of 1 minute. A second reviewer labeled the observed rainfall and wiper state for the largest storm event (2014-08-11) at an enhanced time resolution of roughly 3 seconds. Due to the time-intensive nature of labeling video data at this temporal resolution, and due to the strong agreement between the two labeled datasets, this second round of labeling was not performed for the remaining two days (2014-06-12 and 2014-06-28). Despite the difference in time resolution, manual labeling of the video data showed strong agreement. Taking the high-resolution dataset to represent the ground truth rainfall state (and aggregating the high-resolution dataset to the temporal resolution of the low-resolution dataset), the true positive rate of the low temporal-resolution camera observations was 92.6%, while the true negative rate was 99.3%. Agreement in terms of positive detection was lower due to the difference in temporal resolution between the two sources. The low-resolution camera observation dataset classifies each minute-long interval as either "raining" or "not raining". However, the high-resolution ground truth dataset contains many instances in which part of a given minute-long interval contains rain, and part does not. Thus, when the high-resolution dataset is aggregated to match the resolution of the low-resolution dataset, there are more intervals where some amount of rain is detected (yielding more instances of positive detection overall). A similar mismatch occurs if the low-resolution dataset is interpolated to match the time resolution of the high-resolution dataset. This time resolution mismatch also affects comparisons between the ground truth and other data sources (e.g. wiper, radar and gages). In general, the difference in classification performance between data sources decreases when the ground truth dataset is aggregated in time. Differences in classification performance become more pronounced when a high-resolution ground truth dataset is used.

Many vehicles exhibited data quality issues such as non-reporting wiper sensors, malfunctioning wiper sensors, or unobservable wiper modes. These data quality issues may impact the performance of the wiper as a classifier, but are largely attributable to the fact that the data is taken from a pilot study in which sensor configurations are not standardized. For some vehicles, wiper sensors were simply not configured to report wiper data. In these instances, the reported wiper value was zero for the entire observation period even though wiper movement was observed during manual inspection of the dashboard footage. Vehicles for which wiper sensors were not configured were removed from the analysis. Other vehicles exhibited malfunctioning or poorly configured sensors. For instance, in some cases the wiper intensity fluctuated between 0 and 1 at a frequency on the order of milliseconds—a behavior which is clearly not possible for a human driver. Video footage confirmed that the sensor was malfunctioning during these time periods. Malfunctioning vehicles were also removed from the analysis. Perhaps the most common data quality issue, however, is that several vehicles exhibited unobservable wiper modes. In this case, sensors were configured to report some wiper intensity states but not others. For example, the sensor may report the wiper intensity when the wiper switch is in a "continuous" mode, but may not report the wiper intensity when the wiper is placed in a manual "wipe" mode. These cases could only be detected by manual inspection of the camera footage. These data issues can largely be attributed to the fact that the sensor data is taken from a pilot study in which sensor configurations vary from vehicle to vehicle. As manufacturers standardize sensor configurations for connected vehicles, the relevance of these issues is likely to diminish.

The performance of the wiper as a classifier can be improved by (i) comparing wiper data against a ground truth dataset obtained at a high temporal resolution, and (ii) correcting errors in the wiper sensor readings. When manual observations of the wiper state are used to correct unobservable wiper modes, and the resulting corrected wiper data is compared to the 3-second resolution camera observations, the binary classification performance over weather radar is significantly enhanced: the true positive rate of the wiper data is 5.2% higher than radar, while the true negative rate is 7.7% higher. Table S1 shows the true and false positive rates for all technologies (during the 2014-08-11 storm event) when these two conditions are met.

Fig. S1

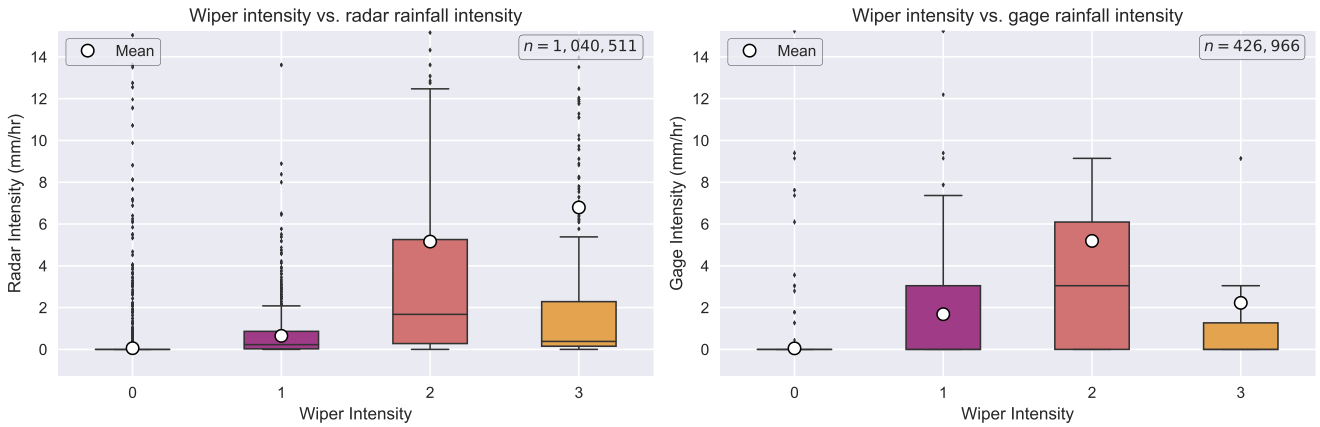

Figure S1. Comparison of radar, gage and wiper intensities for three storm events on 6/12/2014, 6/28/2014, and 8/11/2014. The left panel shows the distribution of radar precipitation measurements associated with each wiper intensity. The right panel shows the distribution of gage precipitation measurements associated with each wiper intensity for vehicles located within 2 kilometers of the gage. Note that the range limitation reduces the number of data points available. No clear relationship is observed between wiper intensity and rainfall intensity.

Table S1

Metric	Gage	Radar	Wiper
True Positive Rate (%)	55.1	91.8	97.0
True Negative Rate (%)	96.9	87.4	95.1

Table S1. Classification performance of each rainfall measurement technology when using high-temporal resolution ground truth data, and correcting misreporting wiper states. These binary performance metrics hold when (i) ground truth observations at a resolution of 2.4 seconds are used, and (ii) manual corrections are made to the wiper state according to the wiper state in the observed camera footage (i.e. unobservable wiper modes are corrected).

Video S1

Original rainfall field (top) vs. updated data product (bottom) for a large storm event on 2014-08-11. Vehicle paths can be seen in the bottom frame, with windshield wiper intensities indicated by greyscale intensity from off (white) to high intensity (dark grey).
